# The HGF/c-MET axis as a potential target to overcome survival signals and improve therapeutic efficacy in multiple myeloma

**DOI:** 10.20517/cdr.2021.73

**Published:** 2021-10-21

**Authors:** Paolo Giannoni, Daniela de Totero

**Affiliations:** ^1^Department of Experimental Medicine, University of Genoa, Genoa 16132, Italy.; ^2^Molecular Pathology Unit, IRCCS Ospedale Policlinico San Martino, Genoa 16132, Italy.

**Keywords:** HGF, c-MET, microenvironment, multiple myeloma, multidrug resistance marker

## Abstract

Multiple myeloma (MM) accounts for about 10% of hematologic malignancies, and it is the second most frequent hematologic neoplasm after lymphomas. The exact etiology of MM is still unknown and, despite the introduction of more effective and safe drugs in recent years, MM remains an incurable disease. Intrinsic and acquired resistance of malignant B cells to pharmacological treatments still represents an obstacle for survival improvement. Activation of the hepatocyte growth factor/c-MET axis has been reported as involved in MM pathogenesis: hepatocyte growth factor (HGF) levels are in fact higher in sera from MM patients than in healthy controls, the HGF/c-MET pathway may be activated in an autocrine or paracrine manner, and it is interesting to note that a higher c-MET phosphorylation is associated with disease progression. Several studies have further demonstrated the over-activation of c-MET either in resistant cell lines or in primary malignant plasma cells purified from bone marrow of patients resistant to chemotherapy. For this reason, c-MET has been proposed as a potential marker of multidrug resistance in the disease. Here, we first summarize the potential role of HGF/c-MET interaction in disease evolution and then describe novel approaches targeting this axis which could be conceptually utilized, alone or in combination with standard therapies, to treat MM and possibly overcome drug resistance.

## INTRODUCTION

Multiple myeloma (MM) is characterized by clonal expansion of malignant plasma cells within bone marrow (BM) and accounts for about 10% of hematological malignancies. Despite a significant improvement in therapies in the last years, the disease remains incurable: a deeper comprehension of the malignant cell biology, in the context of its tumor microenvironment, appears necessary in the attempt to eradicate the disease. The complex interplay between myeloma cells and neighbor cells of the microenvironment is in fact critical in the pathogenesis of MM and drug resistance. Several cellular components of the tumor microenvironment provide support to differentiation and expansion of the malignant clone either directly, by cell-to-cell interaction, or indirectly, through the release of soluble mediators (cytokines, microvescicles, and exosomes). Interleukin-6 (IL-6), released by mesenchymal stem cells (MSCs)^[1]^ has long been recognized as one of the major cytokines involved in the survival and expansion of the malignant clone^[2,3]^. However, IL-6 is also secreted by the malignant plasma cells themselves^[2,3]^, thus suggesting the co-existence of autocrine and paracrine loops supporting myeloma cells proliferation. Many other growth factors, secreted within the microenvironment, have been described as favoring MM cells expansion (i.e., VEGF, MIP1α, BAFF, and IGF-1)^[4]^. Among these, it has been observed that hepatocyte growth factor (HGF) has a critical role in disease progression from monoclonal gammopathy of undetermined significance (MGUS) to MM^[5]^. Indeed, the levels of HGF are 50% higher in MM patients’ sera than in that of healthy controls and correlate with poor prognosis, short-term response to therapies, and early relapse^[6-8]^. The aim of this review is to summarize the key findings on the pathogenetic activity of HGF in multiple myeloma and further describe novel therapeutic approaches potentially helpful to overcome signals provided by the HGF/c-MET axis, putatively involved in drug resistance.

## MALIGNANT PLASMA CELLS RELEASE HGF

The evidence of abnormally high amounts of HGF in sera from MM patients prompted researchers to investigate whether this cytokine could be produced by the malignant plasma cells themselves. Some authors therefore proved that malignant plasma cells secreted HGF and contemporarily expressed its receptor, the tyrosin-kinase c-MET^[9,10]^. HGF mRNA and HGF protein levels appeared higher in MM plasma cells than in plasma cells from normal controls, although with a large variability within the group of examined patients^[9-13]^. It is of further interest to note that HGF production resulted in being increased when MM cells were co-cultured with bone marrow stromal cells (BMSCs), therefore indicating that BMSC-MM cell interaction stimulates HGF release. Furthermore, MM cells expressed and secreted the HGF activator, thus autocatalyzing HGF activation^[14]^; collectively, these findings suggest that HGF-mediated proliferation of malignant plasma cells may also involve an autocrine loop. Aiming to demonstrate the existence of autocrine release and subsequent stimulation of MM growth, Hov *et al.*^[15]^ reported that either a selective c-MET inhibitor (PHA665752) or a neutralizing anti-HGF antibody reduced proliferation of the myeloma cell line ANBL-6 as well as primary MM cells. The supposed autocrine HGF-c/MET growth loop was however more consistently provided for the ANBL-6 cell line, due to a more limited spontaneous proliferation of purified CD138+ primary MM cells.

The observations from Rocci *et al.*^[16]^ further demonstrate that CD138+ cells were characterized by higher c-MET mRNA expression levels than CD138-, when the two fractions were both purified from matched BM of 20 MM patients. Interestingly, the authors also reported that the levels of c-MET expression appeared directly correlated with the response to therapy: patients with lower levels of c-MET mRNA showed a better response.

## HGF AND THE TUMOR MICROENVIRONMENT

In multiple myeloma, neoplastic plasma cells expand, homing into the BM. The BM niche is composed of cells, extracellular matrix proteins, and cytokines. Altogether these components contribute to the induction of a permissive microenvironment leading to enhanced survival, proliferation, migration of neoplastic cells, or resistance to therapy. The demonstration that HGF was constitutively produced by BMSC^[17,18]^ suggests paracrine stimulation of malignant plasma cells. Indeed, functional studies from Derksen *et al*.^[19]^ showed that HGF had strong proliferative and anti-apoptotic effects on MM primary cells or cell lines. Zaman *et al.*^[20]^ further demonstrated, in co-culture studies, that the c-MET inhibitor ARQ197 (tivantinib) could overcome the anti-apoptotic support provided by the stromal NK-tert cells in different MM cell lines (U266 and OPM-2). In addition, HGF was capable of enhancing IL6-mediated proliferation as well as migration of malignant plasma cells, thus suggesting that these two cytokines, both released by BMSC, may synergize and cooperate to induce their expansion^[21]^.

Decorin is a stromal small leucin-rich proteoglycan molecule interacting with HGF. Interestingly, it appeared abnormally low in BM plasma of MGUS or MM patients, as compared with plasma from normal controls^[22]^. The demonstration that decorin inhibited HGF-induced viability and migration of myeloma cell lines *in vitro*^[22] ^suggests that deregulation of the physiological crosstalk among decorin, HGF, and c-MET could have a role in disease pathogenesis. Therefore, the observation that patients with higher levels of decorin in BM plasma showed a better response to therapy supports this suggestion^[23]^. Moreover, low levels of decorin were directly correlated with the presence of osteolytic bone lesions. It has been further demonstrated that malignant plasma cells inhibited osteoblast-induced decorin secretion, while the increase in bone formation had a negative impact on MM cell growth^[24]^. Altogether these observations indicate that the interaction between decorin and cells of the microenvironment (BMSC, osteoblasts, or osteoclasts) might be, directly or indirectly, involved in disease progression. Future investigations are however necessary to better define if the control of MM disease can really be improved through manipulation of the interactions between decorin and the MM niche.

Hepatocyte growth factor also has a pivotal role in angiogenesis, which is a constant hallmark of progression in MM^[25-27]^. Myeloma-induced angiogenesis involves either the direct production of stimulatory molecules by myeloma cells or their induction both in BMSC and in endothelial cells (ECs)^[28]^. Interestingly, Ferrucci *et al.*^[29]^ demonstrated that ECs from BM of patients with MM expressed significantly higher levels of HGF and c-MET, as compared to ECs from normal controls. In addition, the HGF/c-MET pathway appeared constitutively activated in ECs from patients with active disease but not in those derived from patients with MGUS or controls^[29]^. Higher expression of phosphorylated c-MET (p-c-MET) was also detected in relapsed and refractory patients. These findings suggest that the HGF/c-MET autocrine loop is operative in MM-ECs, further confirming the critical role played by this signaling cascade in angiogenesis. The interaction between HGF and c-MET in MM-ECs also mediated their migratory activity, as assayed by a wound scratch or a migratory test, towards VEGF + FGF in the presence of c-MET or HGF inhibitors. These inhibitors further significantly reduced the spontaneous propensity of MM-ECs to spread and form a closely knit capillary network, when seeded on a Matrigel surface^[29]^.

Hepatocyte growth factor also contributes to the regulation of the immunosuppressive state in MM plasma or microenvironmental cells. Bonanno *et al*.^[30]^, in fact, reported that the levels of kynurenine in sera and BM fluid collected from MM patients and the expression of indoleamine 2,3-dioxygenase 1 (IDO1) in neoplastic cells were higher than those in normal controls. Moreover, in MM patients, the subset of CD4+CD25+ FOXP3+Treg cells resulted in being expanded. It is further worth noting that the levels of IDO1 expression directly correlated with HGF amounts and were dramatically increased in PB or BM fluid from patients with MM, as compared with those having MGUS or smoldering myeloma (SMM). Since HGF may be a powerful enhancer of IDO1^[31]^, it is plausible that increased concentrations of HGF, released in either an autocrine or paracrine way, can induce IDO1 upregulation. In support of this statement, the authors indeed demonstrated that the provision of a c-MET kinase inhibitor remarkably downregulated IDO1 expression in U266 and MOLP-8 cell lines.

Collectively, all the data above highlight how HGF, provided by cells of the MM microenvironment, may contribute to enhance survival, expansion, and drug resistance of malignant plasma cells.

## HGF AND THE BONE DISEASE IN MM

About 80%-90% of MM patients develop bone disease^[32,33]^. Cytokines released by MM or microenvironmental cells, directly or indirectly, affect physiological bone remodeling. Different molecules have been identified as involved in bone destruction. However, currently, there is no clear unified theory of how myeloma cells degrade bone tissue. This topic was recently exploited in a comprehensive review by Borset *et al*.^[32]^: here, the authors historically followed the evolution in the knowledge of bone disease in MM patients. They interestingly mentioned that in 1989 Bataille *et al.*^[34]^ observed that malignant cells led to the destruction of the bone tissue through osteoclasts activation, but the disease was also characterized by a reduction in bone formation. These observations thus collectively suggest that MM patients lose the physiological balance in the proper activities of osteoclasts and osteoblasts. Borset *et al.*^[9]^, searching for MM cell-released factors capable of inhibiting cytokines mediating osteoblasts differentiation (i.e., TGFβ), identified HGF as a potential candidate. HGF was then confirmed as involved in MM pathogenesis^[9,35,36]^. The demonstration that HGF was released by osteoclasts, while osteoblasts expressed c-MET, then suggested that HGF might act as a coupling factor between these two cellular types^[37]^. Indeed, Standal *et al.*^[38]^ demonstrated that HGF inhibited osteoblastogenesis induced *in vitro* by bone-morphogenetic protein: a reduced expression of RUNX2 and Osterix in osteo-induced MSC was in fact detected, as well as a decrease in mineralization. These *in vitro* data were further supported by findings of a negative correlation between HGF and bone-specific alkaline phosphatase, a marker of osteoblast activity, in sera from 34 MM patients^[38]^. A positive correlation was instead established between HGF expression in the BM microenvironment of MM patients and the extent of bone disease^[22]^. The demonstration of RANKL upregulation in two stromal cell lines (ST2 and MC3T3-E1) and BMSC, through c-MET/NFKB signaling, has further supported a role for HGF in stimulation of bone remodeling and in osteoclastogenesis. In addition, it has been recently shown that, in osteoblast-like cells, HGF/c-MET signaling could also be mediated by extracellular vesicles (EV) released by myeloma cells^[39]^. In this study, the authors demonstrated that HGF was bound on the surface of EV derived from the HGF-positive MM cell line JJN3, and induced c-MET phosphorylation as well as secretion of the osteoclast-activating cytokine IL-11.

**Figure 1:**
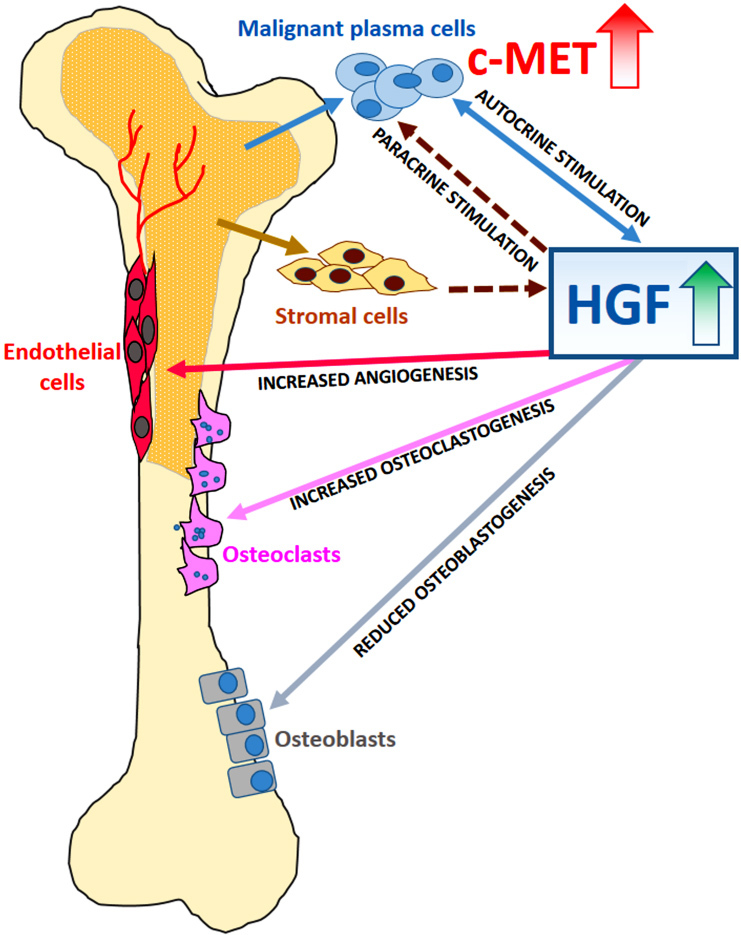
Schematic illustration of HGF-mediated functions on malignant plasma cells or accessory cells of the MM microenvironment. Higher levels of HGF have been detected in sera or BM plasma from MM patients than in that from normal controls. HGF, produced in both autocrine and paracrine manners, may stimulate survival and expansion of MM cells, increase angiogenesis and osteoclastogenesis, and reduce osteoblastogenesis. HGF: Hepatocyte growth factor; MM: multiple myeloma; BM: bone marrow.

The various interactions taking place among MM cells and surrounding microenvironment detailed above are summarized in Figure 1.

## THERAPEUTIC APPROACHES TARGETING HGF/C-MET AXIS TO OVERCOME DRUG RESISTANCE IN MM

Binding of biologically active HGF to c-MET induces receptor dimerization, resulting in trans-phosphorylation of two tyrosine residues (Y1234 and Y1235) in the kinase domain followed by auto-phosphorylation of two tyrosine residues (Y1349 and Y1356) in the C-terminal region. Phosphorylation of Y1349 and Y1356 creates a multi-substrate docking site that is necessary for the induction of downstream signaling cascades such as Ras/Raf/MAPK, PI3K/AKT/mTOR, and STAT3/5 driving cell growth, survival, migration, and angiogenesis^[40,41]^. Inhibition of HGF/c-MET engagement may therefore be helpful in devising novel therapeutic strategies to overcome drug resistance.

### Inhibitor of c-MET tyrosine kinase

A pre-clinical rationale for targeting the c-MET pathway in patients with relapsed and resistant MM was previously suggested in a manuscript by Moschetta *et al.*^[35]^ evaluating the activity of the c-MET inhibitor SU11274 in MM cell lines, either sensitive or resistant to anti-myeloma drugs. They intriguingly observed higher levels of constitutive c-MET and its phosphorylation in resistant cell lines (RPMI8226.R5 and MM.1R) than in their sensitive counterparts. Downmodulation of c-MET and the levels of apoptosis, induced by SU11274, appeared more pronounced in resistant than in sensitive cell lines. Similar results were also obtained with malignant B cells purified from MM patients. It is worth noting that the increase of p-c-MET amount paralleled disease progression and that SU11274 exerted a marked cytotoxic effect on malignant cells from relapsed/resistant patients. Moreover, SU11274 appeared capable of reverting resistance to bortezomib, melphalan, and doxorubicin in RPMI8226.R5 cells. These *in vitro* studies were also validated by an *in vivo* pre-clinical model utilizing NOD/SCID mice xenotransplanted with RPMI8226.R5 cells^[35]^. Altogether these observations are in agreement with data presented in two other manuscripts describing increased sensitivity to bortezomib and doxorubicin of the c-MET siRNA-silenced U266 cell line^[42,43]^. These findings suggest that c-MET overactivation and higher p-c-MET levels could represent a marker of multidrug resistance in MM.

A confirmation of the involvement of c-MET in disease progression was recently provided by Zhang *et al.*^[44]^, through the determination of the prevalence of c-MET overexpression in a publicly available gene expression profiling database of myeloma: c-MET resulted in being significantly higher in MGUS (*n *= 44) *vs.* healthy controls (*n *= 22), and even significantly much higher in a large cohort of newly diagnosed MM cases (*n *= 599), in association with poor outcomes^[44]^. By the use of a new DNA aptamer (SL1), selectively targeting c-MET with high affinity^[45]^, Zhang *et al.*^[44]^ further demonstrated inhibition of growth, adhesion, and migration of malignant plasma cells co-cultured with the stromal HS5 cell line and synergistic effects when used in combination with bortezomib.

ARQ197 (tivantinib), a small molecule, not ATP competitive c-MET inhibitor, also reduced proliferation of MM cell lines and patient-derived CD138+ plasma cells, downregulating c-MET signaling and inhibiting MAPK and PI3K pathways. Tivantinib further appeared equally effective in inducing apoptosis in MM cell lines resistant to melphalan, dexamethasone, bortezomib, or lenalidomide treatment and cells co-cultured with a protective microenvironment or supportive cytokines. The efficacy of tivantinib was also confirmed in pre-clinical studies in mouse models, thus demonstrating inhibition of cell proliferation, downmodulation of c-MET and p-c-MET, and a significant decrement in bone resorption and bone loss^[20,46]^. Based on these encouraging *in vitro* and *in vivo* studies, Baljevic *et al.*^[47]^ conducted a phase II trial with tivantinib in patients with relapsed or relapsed and refractory myeloma whose disease had progressed after 1-4 prior therapy. Results from this study show stable disease (SD) for 36% of patients, but no partial remission or better was obtained. Interestingly, however, HGF plasma levels generally tended to decrease in patients with SD as compared to those who progressed.

In line with the aforementioned observations, cabozantinib (XL-184), a multi-target tyrosine kinase inhibitor suppressing c-Met activation, maintained stable disease in 8 out of 12 patients with relapsed and/or refractory MM in another clinical phase IB study^[48]^.

**Figure 2:**
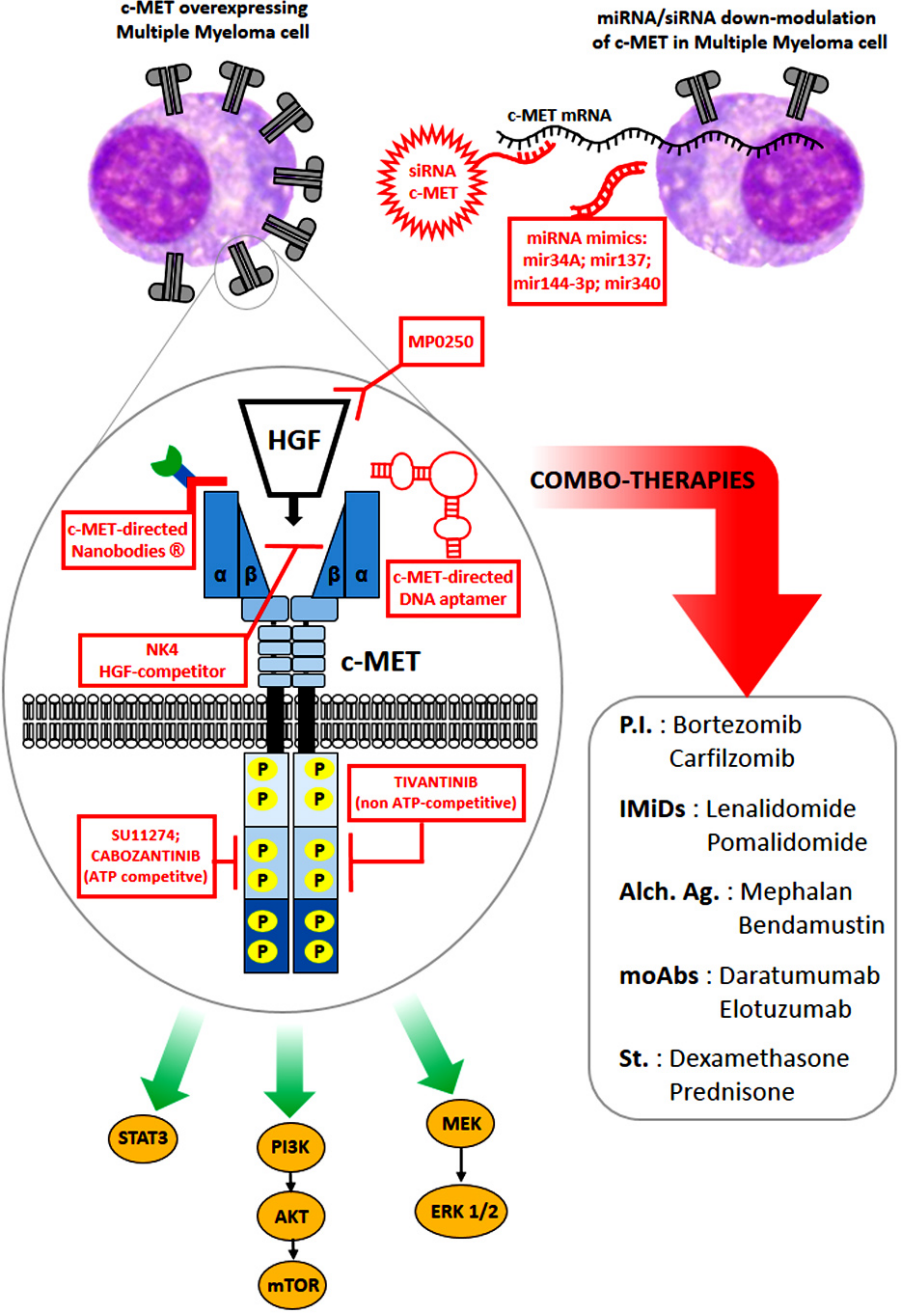
Schematic representation of the potential use of combinatorial therapies exploiting standard treatments [such as PI (proteasome inhibitors), IMiDs (immunomodulatory imide drugs), moAbs (monoclonal antibodies), and St (steroids)] in combination with molecules targeting c-MET or HGF (such as anti-c-MET-tyrosine-kinase inhibitors, neutralizing or competitive anti-HGF antibodies, miRNAs downmodulating c-MET, or siRNA silencing c-MET) to block downstream signals favoring survival, proliferation, and migration of MM cells. HGF: Hepatocyte growth factor; MM: multiple myeloma.

Therefore, although the results of these clinical studies do not seem very promising for the use of anti-c-MET inhibitors as single agents, the authors concluded that it would be rational to consider their use in combination with other anti-myeloma therapeutics, since c-MET can be associated with drug resistance [Figure 2].

### Modulation of miRNA regulating HGF/c-MET activation

A large body of evidence suggests that an imbalance in the expression of several miRNA in plasma cells contribute to MM pathogenesis; those interacting with the HGF/c-MET axis, such as miR-34a, miR-137, and miR-144-3p, appear in general downregulated, as detailed below.

Overexpression of miR-34a has been previously demonstrated to be beneficial in various *in vitro *or *in vivo* models of MM^[49]^; synthetic miR-34a mimics have been therefore proposed as novel therapeutic agents^[50]^. Recently, Li *et al.*^[51]^ reported that, in a xenograft mouse model of MM, scutellarin, a flavonoid glycoside and proteasome inhibitor, reduced disease progression and mitigated chemoresistance to bortezomib through multiple mechanistic pathways involving epigenetic regulation of c-MET/AKT/m-TOR by HDAC/miR-34a, as well as by NF-κB-mediated activation of the apoptotic cascade. Scutellarin/bortezomib co-treatment was significantly more effective than scutellarin or bortezomib alone in downmodulating the different signaling cascades or upregulating miR-34a, further supporting the use of combinatorial therapies.

Moreover miR-137 appeared downmodulated in MM^[52]^. As described by Zhang *et al.*^[52]^, MM cell lines, induced to overexpress miR-137 after its transfection, downregulated c-MET and AKT phosphorylation by targeting the microphthalmia-associated transcription factor (MITF): c-MET is in fact a direct transcriptional target of MITF, and HGF regulates its own receptor levels via MITF. Interestingly, the overexpression of miR-137, or the silencing of MITF by specific siRNA, improved apoptotic rates of MM cells treated with dexamethasone.

In addition, miR-144-3p directly targeted the 3'-untranslated region of c-MET leading to the suppression of c-MET expression and its downstream signaling pathway (PI3K/AKT)^[53]^. Conversely, *in vitro* and *in vivo *(xenografted nude mice) studies demonstrated that restoration of miR-144-3p levels significantly inhibited tumor growth.

Collectively, the above-summarized data indicate that various miRNA-mediated modulations of the HGH/c-MET axis in MM may potentially represent a feasible tool in therapy.

The study by Umezu *et al.*^[54] ^was performed in this direction. Aiming to investigate the therapeutic potential of healthy BMSC-derived exosomes, they described that the high levels of miR-340, present in exosomes purified from young-donor BMSC, strongly inhibited the growth of MM cell lines and CD138+ primary MM cells in an *in vivo* BALB/c nude mouse model. Indeed, miR-340 affected angiogenesis by inhibiting c-MET expression.

### Antibodies competing with HGF or c-MET to block HGF/c-MET axis

Several antagonists of HGF have also been developed trying to block growth, migration, and angiogenesis of MM cells. NK4, composed of the NH2-terminal hairpin loop and the 4 kringle domains of HGF, competes with HGF for c-MET binding. NK4 inhibited the growth of MM cell lines producing HGF both directly (via downmodulation of ERK1/2, STAT3, and AKT-1 pathways) and indirectly via angiogenesis inhibition, as also confirmed in a xenograft mouse model. Moreover, NK4 interestingly enhanced dexamethasone treatment in the KMS11 cell line and overcame resistance to dexamethasone in KMS34-resistant cells. It has been further described that NK4 could also act in non-HGF producing cells, thus suggesting the involvement of an HGF/c-MET independent pathway too^[55]^.

More recently, Rao *et al.*^[56]^ assayed the activity of a MP0250, a multi-domain DARPin® drug that binds simultaneously to VEGF and HGF and thus preventing interaction with their receptors. MP0250 inhibited activation of endothelial cells derived from MM patients (MMEC) by reducing p-VEGF and p-c-MET levels and downstream signaling cascades. MP0250 therefore demonstrated a strong anti-angiogenic effect *in vitro*, showing impairment of chemotaxis and migration and reduced vessel formation. The anti-angiogenic activity of MP0250 has been further confirmed in the 5T33MM syngeneic mouse model, and higher efficiency was detected for the combined MP0250 + bortezomib treatment.

A monovalent anti-c-MET nanobody (small single-domain antibody fragment) was further tested by Slordahl *et al*.^[57]^ as a new approach to block c-MET signals. This nanobody efficiently inhibited HGF-mediated survival, proliferation, migration, and adhesion and reversed HGF-induced inhibition of osteoblastogenesis. Based on its high specificity and potency, with no intrinsic agonistic effect, this nanobody was thus proposed by the authors as a potent and feasible agent for MM treatment.

## CONCLUSION

Among several pathways involved in MM pathogenesis, HGF/c-MET may have a pivotal role. c-MET and its ligand HGF contribute to survival, proliferation, migration, angiogenesis, and bone disease in MM. Findings of abnormal activation of the HGF/c-MET axis in malignant plasma cells, as well as in the MM microenvironment, also in direct correlation with progressive disease, strongly provide support to the use of drugs targeting this pathway to break the crosstalk between myeloma and accessory cells and, possibly, to restore drug sensitivity. Since the results of a limited number of clinical trials seem not too promising when anti-c-MET drugs were used as single agents, we propose that the combination of these molecules with conventional MM therapies could be useful, based also on the observation of c-MET as a potential marker of chemo-resistance.

Therefore, in this prospect, the real challenge could be the availability of reliable techniques capable of evaluating the levels of HGF/c-MET activation and the selection of patients who will benefit from c-MET inhibitors. As summarized above, a wide range of putative inhibitors of the HGF/c-MET axis is currently available and under investigation, such as tyrosine-kinase inhibitors, last-generation antibodies, or even miRNA mimics [Figure 2]. In addition, today novel drugs targeting c-MET or HGF are being developed and tested in ongoing or completed clinical trials in other cancer-cell types^[58]^. Results from these studies could therefore speed up devising novel strategies to overcome drug resistance in MM.

## DECLARATIONS

### Copyright

© The Author(s) 2021.

## References

[1] Kawano M, Hirano T, Matsuda T (1988). Autocrine generation and requirement of BSF-2/IL-6 for human multiple myelomas. Nature.

[2] Borset M, Helseth E, Naume B, Waage A (1993). Lack of IL-1 secretion from human myeloma cells highly purified by immunomagnetic separation. Br J Haematol.

[3] Klein B, Zhang XG, Jourdan M (1989). Paracrine rather than autocrine regulation of myeloma-cell growth and differentiation by interleukin-6. Blood.

[4] Mahindra A, Hideshima T, Anderson KC (2010). Multiple myeloma: biology of the disease. Blood Rev.

[5] Hofmann JN, Landgren O, Landy R (2020). A prospective study of circulating chemokines and angiogenesis markers and risk of multiple myeloma and its precursor. JNCI Cancer Spectr.

[6] Iwasaki T, Hamano T, Ogata A, Hashimoto N, Kitano M, Kakishita E (2002). Clinical significance of vascular endothelial growth factor and hepatocyte growth factor in multiple myeloma. Br J Haematol.

[7] Seidel C, Børset M, Turesson I, Abildgaard N, Sundan A, Waage A (1998). Elevated serum concentrations of hepatocyte growth factor in patients with multiple myeloma. The Nordic Myeloma Study Group. Blood.

[8] Turesson I, Abildgaard N, Ahlgren T (1999). Prognostic evaluation in multiple myeloma: an analysis of the impact of new prognostic factors. Br J Haematol.

[9] Börset M, Hjorth-Hansen H, Seidel C, Sundan A, Waage A (1996). Hepatocyte growth factor and its receptor c-met in multiple myeloma. Blood.

[10] Borset M, Lien E, Espevik T, Helseth E, Waage A, Sundan A (1996). Concomitant expression of hepatocyte growth factor/scatter factor and the receptor c-MET in human myeloma cell lines. J Biol Chem.

[11] Børset M, Seidel C, Hjorth-Hansen H, Waage A, Sundan A (1999). The role of hepatocyte growth factor and its receptor c-Met in multiple myeloma and other blood malignancies. Leuk Lymphoma.

[12] Mahtouk K, Tjin EP, Spaargaren M, Pals ST (2010). The HGF/MET pathway as target for the treatment of multiple myeloma and B-cell lymphomas. Biochim Biophys Acta.

[13] Rampa C, Tian E, Våtsveen TK (2014). Identification of the source of elevated hepatocyte growth factor levels in multiple myeloma patients. Biomark Res.

[14] Tjin EP, Derksen PW, Kataoka H, Spaargaren M, Pals ST (2004). Multiple myeloma cells catalyze hepatocyte growth factor (HGF) activation by secreting the serine protease HGF-activator. Blood.

[15] Hov H, Holt RU, Rø TB (2004). A selective c-met inhibitor blocks an autocrine hepatocyte growth factor growth loop in ANBL-6 cells and prevents migration and adhesion of myeloma cells. Clin Cancer Res.

[16] Rocci A, Gambella M, Aschero S (2014). MET dysregulation is a hallmark of aggressive disease in multiple myeloma patients. Br J Haematol.

[17] Giannoni P, Scaglione S, Quarto R (2011). An interaction between hepatocyte growth factor and its receptor (c-MET) prolongs the survival of chronic lymphocytic leukemic cells through STAT3 phosphorylation: a potential role of mesenchymal cells in the disease. Haematologica.

[18] Takai K, Hara J, Matsumoto K (1997). Hepatocyte growth factor is constitutively produced by human bone marrow stromal cells and indirectly promotes hematopoiesis. Blood.

[19] Derksen PW, de Gorter DJ, Meijer HP (2003). The hepatocyte growth factor/Met pathway controls proliferation and apoptosis in multiple myeloma. Leukemia.

[20] Zaman S, Shentu S, Yang J (2015). Targeting the pro-survival protein MET with tivantinib (ARQ 197) inhibits growth of multiple myeloma cells. Neoplasia.

[21] Hov H, Tian E, Holien T (2009). c-Met signaling promotes IL-6-induced myeloma cell proliferation. Eur J Haematol.

[22] Kristensen IB, Pedersen L, Rø TB (2013). Decorin is down-regulated in multiple myeloma and MGUS bone marrow plasma and inhibits HGF-induced myeloma plasma cell viability and migration. Eur J Haematol.

[23] Huang SY, Lin HH, Yao M (2015). Higher decorin levels in bone marrow plasma are associated with superior treatment response to novel agent-based induction in patients with newly diagnosed myeloma - a retrospective study. PLoS One.

[24] Li X, Pennisi A, Yaccoby S (2008). Role of decorin in the antimyeloma effects of osteoblasts. Blood.

[25] Bussolino F, Di Renzo MF, Ziche M (1992). Hepatocyte growth factor is a potent angiogenic factor which stimulates endothelial cell motility and growth. J Cell Biol.

[26] Vacca A, Ribatti D, Roncali L (1994). Bone marrow angiogenesis and progression in multiple myeloma. Br J Haematol.

[27] Zhang YW, Su Y, Volpert OV, Vande Woude GF (2003). Hepatocyte growth factor/scatter factor mediates angiogenesis through positive VEGF and negative thrombospondin 1 regulation. Proc Natl Acad Sci U S A.

[28] Jakob C, Sterz J, Zavrski I (2006). Angiogenesis in multiple myeloma. Eur J Cancer.

[29] Ferrucci A, Moschetta M, Frassanito MA (2014). A HGF/cMET autocrine loop is operative in multiple myeloma bone marrow endothelial cells and may represent a novel therapeutic target. Clin Cancer Res.

[30] Bonanno G, Mariotti A, Procoli A (2012). Indoleamine 2,3-dioxygenase 1 (IDO1) activity correlates with immune system abnormalities in multiple myeloma. J Transl Med.

[31] Rutella S, Bonanno G, Procoli A (2006). Hepatocyte growth factor favors monocyte differentiation into regulatory interleukin (IL)-10++IL-12low/neg accessory cells with dendritic-cell features. Blood.

[32] Børset M, Sundan A, Waage A, Standal T (2020). Why do myeloma patients have bone disease?. Blood Rev.

[33] Eda H, Santo L, David Roodman G, Raje N

[34] Bataille R, Chappard D, Marcelli C (1989). Mechanisms of bone destruction in multiple myeloma: the importance of an unbalanced process in determining the severity of lytic bone disease. J Clin Oncol.

[35] Moschetta M, Basile A, Ferrucci A (2013). Novel targeting of phospho-cMET overcomes drug resistance and induces antitumor activity in multiple myeloma. Clin Cancer Res.

[36] Rø TB, Holien T, Fagerli UM (2013). HGF and IGF-1 synergize with SDF-1α in promoting migration of myeloma cells by cooperative activation of p21-activated kinase. Exp Hematol.

[37] Grano M, Galimi F, Zambonin G (1996). Hepatocyte growth factor is a coupling factor for osteoclasts and osteoblasts in vitro. Proc Natl Acad Sci U S A.

[38] Standal T, Abildgaard N, Fagerli UM (2007). HGF inhibits BMP-induced osteoblastogenesis: possible implications for the bone disease of multiple myeloma. Blood.

[39] Strømme O, Psonka-Antonczyk KM, Stokke BT, Sundan A, Arum CJ, Brede G (2019). Myeloma-derived extracellular vesicles mediate HGF/c-Met signaling in osteoblast-like cells. Exp Cell Res.

[40] Comoglio PM, Trusolino L, Boccaccio C (2018). Known and novel roles of the MET oncogene in cancer: a coherent approach to targeted therapy. Nat Rev Cancer.

[41] Organ SL, Tsao MS (2011). An overview of the c-MET signaling pathway. Ther Adv Med Oncol.

[42] Que W, Chen J (2011). Knockdown of c-Met inhibits cell proliferation and invasion and increases chemosensitivity to doxorubicin in human multiple myeloma U266 cells in vitro. Mol Med Rep.

[43] Que W, Chen J, Chuang M, Jiang D (2012). Knockdown of c-Met enhances sensitivity to bortezomib in human multiple myeloma U266 cells via inhibiting Akt/mTOR activity. APMIS.

[44] Zhang Y, Gao H, Zhou W (2018). Targeting c-met receptor tyrosine kinase by the DNA aptamer SL1 as a potential novel therapeutic option for myeloma. J Cell Mol Med.

[45] Ueki R, Sando S (2014). A DNA aptamer to c-Met inhibits cancer cell migration. Chem Commun (Camb).

[46] Lath DL, Buckle CH, Evans HR (2018). ARQ-197, a small-molecule inhibitor of c-Met, reduces tumour burden and prevents myeloma-induced bone disease in vivo. PLoS One.

[47] Baljevic M, Zaman S, Baladandayuthapani V (2017). Phase II study of the c-MET inhibitor tivantinib (ARQ 197) in patients with relapsed or relapsed/refractory multiple myeloma. Ann Hematol.

[48] Lendvai N, Yee AJ, Tsakos I (2016). Phase IB study of cabozantinib in patients with relapsed and/or refractory multiple myeloma. Blood.

[49] Misso G, Zarone MR, Lombardi A (2019). miR-125b upregulates miR-34a and sequentially activates stress adaption and cell death mechanisms in multiple myeloma. Mol Ther Nucleic Acids.

[50] Di Martino MT, Leone E, Amodio N (2012). Synthetic miR-34a mimics as a novel therapeutic agent for multiple myeloma: in vitro and in vivo evidence. Clin Cancer Res.

[51] Li L, Zheng Y, Zhang W, Hou L, Gao Y (2020). Scutellarin circumvents chemoresistance, promotes apoptosis, and represses tumor growth by HDAC/miR-34a-mediated down-modulation of Akt/mTOR and NF-κB-orchestrated signaling pathways in multiple myeloma. Int J Clin Exp Pathol.

[52] Zhang B, Ma L, Wei J (2016). miR-137 suppresses the phosphorylation of AKT and improves the dexamethasone sensitivity in multiple myeloma cells via targeting MITF. Curr Cancer Drug Targets.

[53] Zhao Y, Xie Z, Lin J, Liu P (2017). MiR-144-3p inhibits cell proliferation and induces apoptosis in multiple myeloma by targeting c-Met. Am J Transl Res.

[54] Umezu T, Imanishi S, Azuma K (2017). Replenishing exosomes from older bone marrow stromal cells with miR-340 inhibits myeloma-related angiogenesis. Blood Adv.

[55] Du W, Hattori Y, Yamada T (2007). NK4, an antagonist of hepatocyte growth factor (HGF), inhibits growth of multiple myeloma cells: molecular targeting of angiogenic growth factor. Blood.

[56] Rao L, De Veirman K, Giannico D (2018). Targeting angiogenesis in multiple myeloma by the VEGF and HGF blocking DARPin^®^ protein MP0250: a preclinical study. Oncotarget.

[57] Slørdahl TS, Denayer T, Moen SH (2013). Anti-c-MET Nanobody - a new potential drug in multiple myeloma treatment. Eur J Haematol.

[58] Wood GE, Hockings H, Hilton DM, Kermorgant S (2021). The role of MET in chemotherapy resistance. Oncogene.

